# Practical Implementation, Characterization and Applications of a Multi-Colour Time-Gated Luminescence Microscope

**DOI:** 10.1038/srep06597

**Published:** 2014-10-13

**Authors:** Lixin Zhang, Xianlin Zheng, Wei Deng, Yiqing Lu, Severine Lechevallier, Zhiqiang Ye, Ewa M. Goldys, Judith M. Dawes, James A. Piper, Jingli Yuan, Marc Verelst, Dayong Jin

**Affiliations:** 1 Advanced Cytometry Labs, ARC Centre of Excellence for Nanoscale BioPhotonics (CNBP), Macquarie University, Sydney, NSW 2109, Australia; 2 Centre d'Élaboration de Matériaux et d'Etudes Structurales (CERMES - CNRS), Paul Sabatier University, France; 3 State Key Laboratory of Fine Chemicals, School of Chemistry, Dalian University of Technology, Dalian 116024, China

## Abstract

Time-gated luminescence microscopy using long-lifetime molecular probes can effectively eliminate autofluorescence to enable high contrast imaging. Here we investigate a new strategy of time-gated imaging for simultaneous visualisation of multiple species of microorganisms stained with long-lived complexes under low-background conditions. This is realized by imaging two pathogenic organisms (*Giardia lamblia* stained with a red europium probe and *Cryptosporidium parvum* with a green terbium probe) at UV wavelengths (320–400 nm) through synchronization of a flash lamp with high repetition rate (1 kHz) to a robust time-gating detection unit. This approach provides four times enhancement in signal-to-background ratio over non-time-gated imaging, while the average signal intensity also increases six-fold compared with that under UV LED excitation. The high sensitivity is further confirmed by imaging the single europium-doped Y_2_O_2_S nanocrystals (150 nm). We report technical details regarding the time-gating detection unit and demonstrate its compatibility with commercial epi-fluorescence microscopes, providing a valuable and convenient addition to standard laboratory equipment.

Autofluorescence in biological samples presents a universal challenge for conventional fluorescence techniques to detect or visualize target species^1^,^2^,^3^,^4^,^5^,^6^. The time-gated luminescence (TGL) technique, which takes advantages of long-lived luminescent probes (e.g. lanthanide complexes) and time-delayed detection, substantially enhances the signal-to-noise ratio and contrast by suppressing the autofluorescence contribution^7^,^8^,^9^,^10^,^11^,^12^. However, the presently reported TGL microscopes can only image one colour at a time, due to the gating schemes and/or imaging devices that only allow monochromatic visualization^8^,^13^,^14^,^15^,^16^,^17^. Though multiple colours can be superimposed via image processing^18^,^19^, it requires frequent changing of filters and reduces the efficiency when examining different species, as well as limits the opportunity to investigate their interactions. Meanwhile, the previous TGL microscopes required costly components and sophisticated assembly, which are often inaccessible in most chemical and biological laboratories^20^,^21^,^22^,^23^,^24^. These issues have been hindering broad implementation of the time-gated imaging technique.

In our previous work, we demonstrated a low-cost true-colour TGL microscope, featuring an ultraviolet light-emitting diode (UV LED) for excitation and a mechanical chopper for time-gating^13^,^25^,^26^. In order to simultaneously excite multiple long-lived probes, especially terbium with sensitizing moiety complex that needs a triplet energy around 30,000 cm^−1^ to pump Tb^3+^ to its excited state (^5^D_4_; 20,400 cm^−1^)^25^, efficient excitation at 300–340 nm is required; however, the power of currently available UV LEDs at this wavelength range is not strong enough. On the other hand, flash lamps emitting deep UV with high power but low repetition rate (less than 100 Hz) have been used for TGL microscopy^27^,^28^, but the long detection windows prevent efficient collection of lanthanide luminescence typically with decay lifetimes of ~1 ms or less, as well as having stability issues when synchronized to mechanical choppers.

In this work, we report a highly efficient TGL microscopy system for dual-colour low-background imaging. It uses a high-power flash lamp that can be externally triggered at high repetition rate (up to 1 kHz). This system also comprises a purpose designed and optimized time-gating unit that can be simply inserted into a commercial epi-fluorescence microscope. We apply the new imaging system for simultaneous detection of both *Giardia lamblia* stained with a red-emitting europium complex probe and *Cryptosporidium parvum* stained with a green-emitting terbium complex probe against strong autofluorescent background. Sufficient sensitivity of this combined system is demonstrated by imaging single nanoparticles.

## Results

To set up the multi-colour TGL microscope, we built an illuminator featuring a new-generation ceramic xenon flash lamp (FX-4400, Excelitas Technologies) capable of delivering high-power light pulses to excite the sample, and a time-gating unit incorporating a modified mechanical chopper to discriminate long-lived luminescence against rapidly decaying autofluorescence. Both modules were designed and engineered in such a way that they can be directly coupled onto commercial microscopes, for example the Olympus IX71 epi-fluorescence microscope used in our study, to perform TGL imaging with minimum effort required for configuration and alignment.

### Optical layout

**Figure 1:**
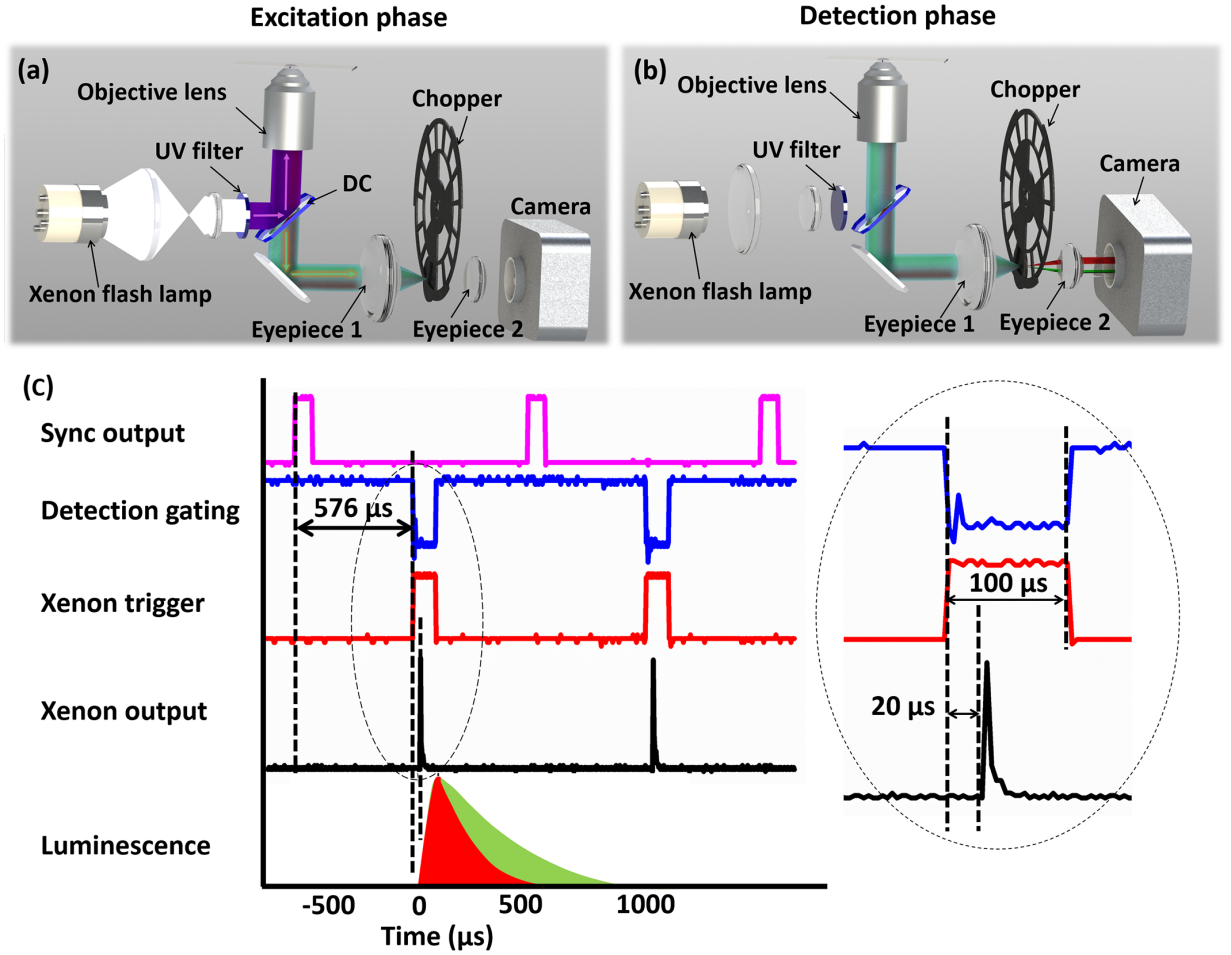
Schematic diagrams of the multi-colour TGL microscope. (a) In the excitation phase, a pulsed excitation light illuminates the sample, while the chopper stops the luminescence/autofluorescence being captured by camera. (b) In the detection phase, the excitation is turned off, and the chopper allows the luminescence to reach the camera. (c) The time sequence of the system is shown, with every repetition cycle containing a gating window of 88 μs and a detection window of 968 μs. Each flash pulse is released 20 μs after the trigger and last around 17 μs.

The schematic diagram of this multi-colour TGL microscope is shown in Figure 1a and 1b. In the excitation phase (Figure 1a), the beam from the xenon lamp passes through a UV band-pass filter (U-360, Edmund), and is reflected by a dichroic mirror (DC shown in Figure 1; 400DCLP, Chroma). Then, it is focused through an objective lens (60×, NA 0.75, Edmund), onto a microscopic slide to excite the specimen. The generated luminescence is collected by the same objective. It is separated from the excitation path by the dichroic mirror, and coupled to the time-gating unit consisting of the chopper, two eyepieces and a digital colour camera (DP71, Olympus). Two eyepieces (Eyepiece 1: ×10, Olympus; Eyepiece 2: RKE 32 mm, wide angle, Edmund) are used to bring down the size of the emission beam, so that the chopper can be placed at the focal spot to block the emission with minimised dead time during the periods of pulsed excitation^13^. When the excitation is off, a short time delay is applied to allow the prompt fading of the autofluorescence background. Therefore, in the detection phase, only the luminescence from the long lifetime lanthanide probes is captured by the camera (Figure 1b). The synchronisation between excitation phase and detection phase should be carefully and accurately carried out. The time sequence used is given in Figure 1c, and the details of synchronisation are described below.

### Excitation source

In this system, the ceramic xenon flash lamp used outputs an average power of 60 W over its full spectrum (from 160 nm to 2000+ nm), and more importantly, a high repetition rate up to 1 kHz. It was coupled into the back port of the IX71 microscope, replacing the original mercury lamp and connecting the collimator using a customized adaptor. In order to minimise the optical background and photo-bleaching of the sample, a UV band-pass filter was used to only select the spectral region which contributed to the excitation of lanthanide probes (320–400 nm; see Supplementary Figure S1). The average excitation power entering the rear aperture of the objective lens was measured to be 2.7 mW. It had a reasonably uniform distribution over a sample area of 200 μm in diameter, leading to an excitation intensity of 8.6 W/cm^2^.

For comparison, our previously reported system using a UV LED (UVTOP310, 315 ± 15 nm; Sensor Electronic Technology) for excitation^25^ was also investigated for multi-colour TGL imaging. Its average excitation power at the rear aperture of the objective lens was measured to be 0.5 mW, hence an excitation intensity of 1.6 W/cm^2^.

### Chopper modification

In order to achieve optimum performance and compatibility with the xenon flash lamp, we modified a high-speed mechanical chopper (C995, Terahertz Technologies Inc). Originally, this device had 30 blades with a fixed duty cycle of 1:1, and it was able to run at a frequency up to 5 kHz. Considering that the highest frequency of the xenon lamp was 1 kHz and the lifetimes of lanthanide probes were in the range of tens to hundreds of μs, we removed 25 blades using ultrafast laser micromachining and only kept one blade out of every six, so that the duty cycle became 1:11 (see Supplementary Figure S2). It is worth mentioning that the reduced weight of the chopper blades also helped reduce the vibration of the time-gating unit to some degree.

A plate with a 1-mm-diameter pinhole was attached to the chopper enclosure to increase the chopping efficiency by removing stray light, as well as protecting the device from dust. The highest chopping frequency after modification was measured to be 947 Hz, which yielded 88 μs for gating and 968 μs for detection.

### Time-gating unit

**Figure 2:**
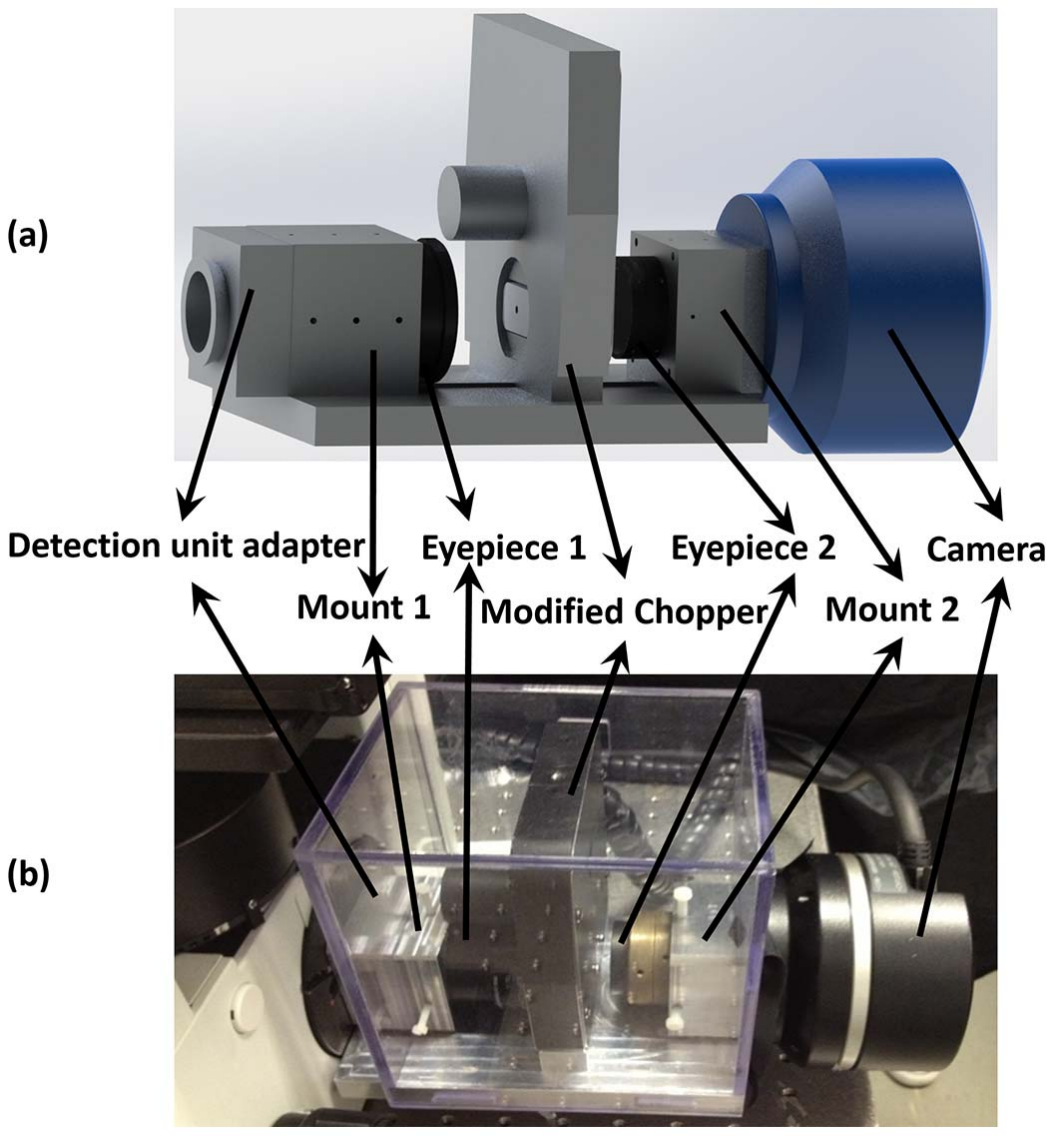
The time-gating unit consists of two eyepieces, one chopper and one camera, all of which are mounted on a common frame. Top: the schematics; Bottom: a photo of the real system.

Incorporating the modified chopper, a time-gating unit was designed and built, as shown in Figure 2. An aluminium frame was machined to mount the eyepieces, the chopper and the camera, alongside an adaptor for the camera port of a standard microscope, in our case the Olympus IX71. Fine alignment of the components was conducted in the bright-field mode illuminated by a halogen lamp. In the first step, the pair of eyepieces was adjusted to make their focuses completely overlap. Since the eyepieces had the same magnification, this was verified by measuring the beam size at a long distance (e.g. 1 m) away along the optical path, which should remain identical regardless of the presence/absence of the two eyepieces. In the second step, the modified chopper was inserted between the eyepieces, so that its pinhole plate was located exactly at the common focus of the two eyepieces. In the third step, the camera was mounted after the second eyepiece. The imaging quality of the system was examined using a microscopic reticle (grid distortion targets, Thorlabs). The most common optical aberration encountered during the alignment was the “barrel distortion” (see Supplementary Figure S3); however, this could be effectively overcome once all optical components were adjusted to be exactly coaxial. After proper alignment, the obtained field-of-view was 150 × 150 μm^2^.

### Synchronisation

After all the components were properly aligned, synchronisation between the excitation and the time-gated detection was elaborately carried out to ensure maximum contrast enhancement for TGL imaging. As shown in Figure 1c, we first measured the time delay between the *Sync Output* from the chopper controller (which was generated by a built-in sensor in the chopper head for monitoring the rotation of the blades) and the physical opening/blockage of the detection path. The latter was recorded by a photodiode placed after the second eyepiece when the bright-field illumination was switched on. With the modified chopper blades, this delay from the rising edge of *Sync Output* to the blockage of the detection path was determined as 576 μs, which was essentially caused by the different positions of the sensor and the pinhole. Then, we fed the *Sync Output* from the chopper controller to a digital delay generator (DG535, Stanford Research Systems), which sent TTL pulses of 100 μs duration to trigger the xenon lamp. Because of the gas discharge process, there was another time delay from the rising edge of the trigger signal to the time of flashing, which was measured as 20 μs. Finally, we adjusted the delay value between the *Sync Output* channel and the xenon trigger channel while monitoring the level of autofluorescence leakage using a piece of paper as the reference sample. It was found that a delay value of 576 μs (same as the delay between *Sync Output* and chopper blockage) rendered the autofluorescence invisible and allowed the maximum luminescence signal to be captured.

### Dual-colour imaging

**Figure 3:**
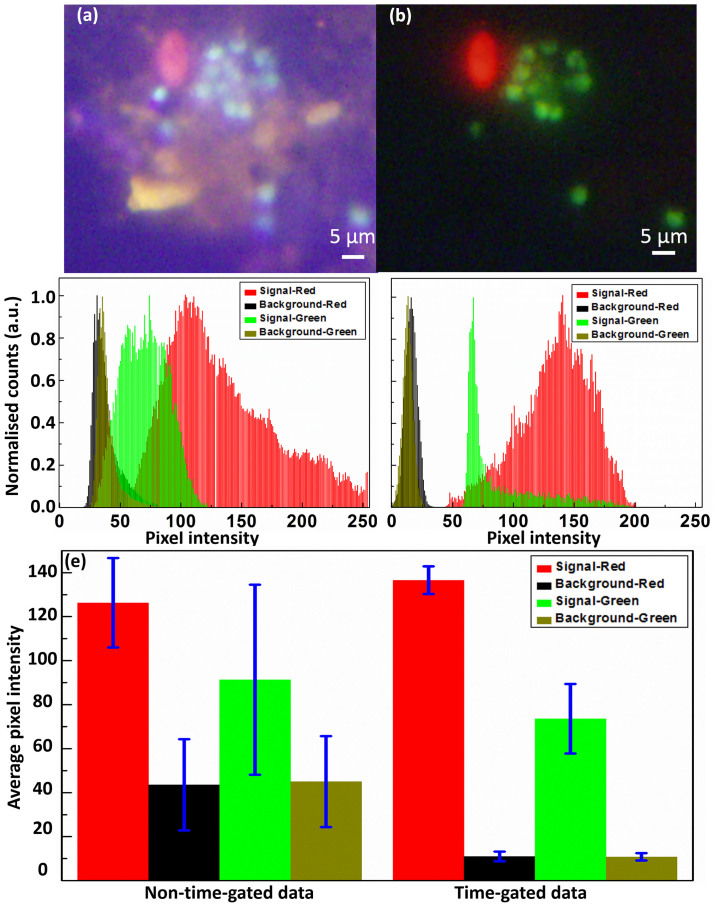
Two-colour imaging under xenon lamp excitation. (a) and (b) are non-time-gated and time-gated images of *Giardia lamblia* cysts labelled with a red europium probe and *Cryptosporidium parvum* oocysts labelled with a green terbium probe (exposure time: 5 seconds). (c) and (d) show the pixel intensity histograms for the signal area (target cells) and the background area in separate red and green channels for (a) and (b), respectively. (e) is a bar chart showing the average signal and background levels summarised from 10 pairs of non-time-gated and time-gated dual-colour images. Error bars represent ±1 s.e.m.

Two waterborne pathogens, *Giardia lamblia and Cryptosporidium parvum*, were labelled with Eu and Tb luminescent complexes, respectively (see Methods for details). The mixed samples were imaged under xenon flash lamp excitation, with the non-time-gated result shown in Figure 3a and the time-gated result shown in Figure 3b for one typical sample area. To evaluate the signal-to-background ratios in an accurate and objective way, the intensity levels of targets as well as the rest areas were carefully analysed in the separate red and green channels (detailed procedures see Supplementary Information S4). As shown in Figure 3c and 3d, the part of signals that were once submerged under the background clearly stood out after the time-gated mode was applied. The average values given in Figure 3e, after statistically measuring 10 pairs of non-time-gated and time-gated images (complete data see Supplementary Table S1 and S2), indicate the signal-to-background ratio is enhanced by 4.3-folds (from 126:44 to 137:11) for the red channel, as well as 3.3-folds (from 91:45 to 74:11) for the green channel, with camera exposure time of 5 seconds. By contrast, previously it took 15 seconds (repetition rate 2.5 kHz, excitation pulse 100 μs, detection window 300 μs) to accumulate enough luminescent signals (average 70 for red and 37 for green) under 315 nm UV LED excitation (see Supplementary Information S5). Therefore, it is calculated that the excitation efficiency of the xenon lamp is 5.9 times in red and 6.0 times in green higher than that of the UV LED, thus more suitable for multi-colour TGL microscopy.

### Crosstalk between red (Eu) and green (Tb) channels

We proposed a calibration method to calculate the crosstalk from the emission spectra of lanthanide chelates and the responsivity curves of the true-colour DP71 camera (detailed procedures see Supplementary Information S6). Briefly, 78.6% of the total emission from the Eu complexes is collected into the red channel of the camera, along with 13.6% and 7.8% into green and blue channels, respectively. Meanwhile, for the Tb complexes, since its emission spectrum has satellite peaks in the blue and red range, 59.9% of the total emission is collected into the green channel, 19.2% into the red channel, and 20.9% into the blue channel. These values can be applied to compensate the original imaging results to achieve more precise quantitative measurement. However, for applications that aim to detect target cells or analytes only, the crosstalk issue may be ignored if the contrast in time-gated images is sufficiently high, as was the case in this study.

### Single nanoparticle sensitivity

**Figure 4:**
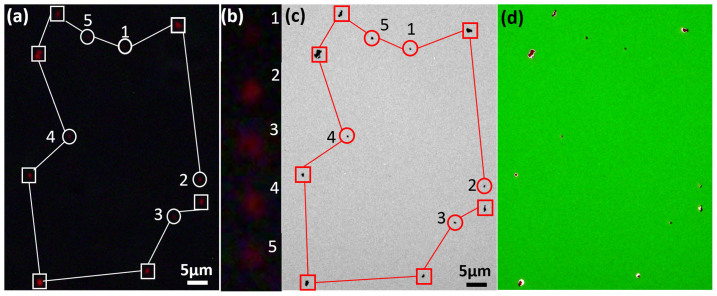
Imaging results of the Y_2_O_2_S:Eu nanoparticles. (a) A time-gated image shows a pattern of luminescence spots. The spots marked with circles (numbered as 1 to 5) contain single Eu nanoparticles, while the others marked with squares contain aggregation of nanoparticles. (b) The enlarged images are given for spots 1–5. (c) The TEM image shows the same pattern for the spots of nanoparticles. (d) Co-localisation analysis confirms the perfect match between the time-gated image and the TEM image.

We further evaluated the sensitivity of the new time-gated luminescence microscope by imaging single nanoparticles. Figure 4 presents a typical result of 150 nm Y_2_O_2_S:Eu^3+^ nanoparticles under xenon lamp excitation with an exposure time of 30 seconds. While the non-time-gated mode failed to provide enough contrast (Figure not shown), the time-gated mode offered sufficient sensitivity to observe these nanoparticles down to a single one (Figure 4a). Figure 4b enlarges images of the luminescent spots which potentially contain single nanoparticles (others were easily to be identified as aggregation of nanoparticles). Following a literature method^14^,^29^, they were eventually confirmed in the corresponding transmission electron microscopy (TEM) image (Figure 4c), which showed perfect correlation with the luminescence image (Figure 4d; detailed procedures see Supplementary Information S7).

## Discussion and Conclusion

In this study, we demonstrate for the first time that simultaneous dual-colour visualisation can be realised in TGL imaging for two species of microorganisms stained with different lanthanide probes. We show that outstanding sensitivity and contrast can be achieved with a new-generation xenon flash lamp that is capable of pulsed excitation at high repetition rate. Our compact time-gating unit provides an easy-to-use and low-cost option to chemists developing lanthanide materials and biologists who wish to eliminate autofluorescence background. Furthermore, the system is also compatible to the automated scanning and lifetime measurement techniques^30^,^31^, to enable high-throughput detection and analysis of multiple target microorganisms. With the rapid progress in lanthanide chemistry, especially the new development of lifetime-tunable lanthanide probes^32^, we believe such multi-colour TGL technique will have a broad range of impact on analytical and bio-sensing applications.

## Methods

### Immunoluminescent staining of Giardia lamblia

Immunofluorescence staining of *Giardia lamblia* was carried out based on the published method^24^ with slight modifications. 30 μL of mouse monoclonal anti-*Giardia* antibody G203 (IgG, cyst-wall specific, 0.44 mg/mL, BTF-bioMérieux), 100 μL of biotinylated goat anti-mouse IgG antibody (1:10 dilution; Catalogue Number AP200B, ChemiCon International, Millipore Bioscience Division), and 100 μL of SA-BSA-BHHCT-Eu^3+^ conjugate (50 μg/mL) (the synthesis method was reported in literature^13^) were mixed with 10 μL suspension of *Giardia lamblia* (containing ~2,500 *Giardia lamblia* cysts, BTF-bioMérieux), and incubated at room temperature for 12 hours. In order to separate the Eu-labelled cysts, the FACSAria flow cytometer (Becton Dickinson) was used to sort out the sample prepared above, which yielded ~2,000 stained cysts in 400 μL 1.25% PBS solution.

### Immunoluminescent staining of Cryptosporidium parvum

For staining of *Cryptosporidium parvum*, a method similar to that reported in literature^33^ was used. In a typical procedure, 10 μL suspension of *Cryptosporidium parvum* oocysts (~10^6^ oocysts/mL in PBS, BTF-bioMérieux) was mixed with 20 μL of mouse monoclonal anti-Cryptosporidium antibody C104 (IgG, 0.44 mg/mL; BTF-bioMérieux) and incubated for 24 hours at room temperature. The mixture was then centrifuged (at 12,000 rpm, 5 minutes) and washed with PBS (pH 7.2) three times. After removing the supernatant, 20 μL of 10-fold diluted biotinylated goat anti-mouse IgG antibody was added and incubated for another 24 hours at room temperature, followed by washing and centrifugation. Subsequently, 20 μL of the Tb-labelled streptavidin (1:20 dilution of 1 mg/mL kit, LanthaScreen® Tb-Streptavidin, Invitrogen) was added and the suspension was incubated for another 48 hours. Finally, the stained *Cryptosporidium* oocysts were washed three times with PBS.

### Preparation of mixed pathogen samples

In order to demonstrate the background suppression feature of our system, a sample exhibiting strong autofluorescence was prepared. Flower petals with different colours of native chromophores were pulverized and mixed with fruit juice, which is also known to be autofluorescent. They were filtered through a centrifugal filter device (UFC30GV00, Millipore) to remove large fragments.

The two stained microorganisms and artificial background samples were mixed and sandwiched between a glass slide and a coverslip. 5 μL of background solution was dropped onto a glass slide. After it dried, 2 μL of the Eu-probe-labelled *Giardia lamblia* cysts was added. 10 minutes later, 2 μL of the Tb-probe-labelled *Cryptosporidium parvum* was dropped on top of the sample, and the whole preparation was covered with a coverslip.

### Preparation of single nanoparticle samples

10 μL of an ethanol solution containing 0.25 mg/mL Eu-doped nanoparticles Y_2_O_2_S:Eu^3+^ (5% dopants, average size 150 nm)^34^ was dropped on the copper grids coated by amorphous carbon. The nanoparticles on the grid were imaged by a transmission electron microscope (TEM, Philips CM10).

## Author Contributions

Y.L. and D.J. conceived the project, designed the experiments and supervised the research. L.Z., X.Z. and D.J. were primarily responsible for microscopy setups, evaluation, data collection and analysis. L.Z., X.Z., Y.L. and D.J. prepared figures and wrote the main manuscript text. W.D., S.L., J.Y., Z.Y. and M.V. provided the samples and conducted image data analysis. J.M.D., E.M.G. and J.A.P. contributed to data analysis and manuscript preparation.

## Supplementary Material

Supplementary Information Practical Implementation, Characterization and Applications of a Multi-Colour Time-Gated Luminescence Microscope

## References

[b1] Gahlaut N. & Miller L. W. Time-resolved microscopy for imaging lanthanide luminescence in living cells. Cytom Part A 77A, 1113–1125, 10.1002/Cyto.A.20964 (2010). 10.1002/cyto.a.20964 PMC3076717 20824630

[b2] Tian L. *et al.* Preparation and time-gated luminescence bioimaging applications of long wavelength-excited silica-encapsulated europium nanoparticles. Nanoscale 4, 3551–3557, 10.1039/C2nr30233k (2012). 22552488 10.1039/c2nr30233k

[b3] Rich R. M. *et al.* Elimination of autofluorescence background from fluorescence tissue images by use of time-gated detection and the AzaDiOxaTriAngulenium (ADOTA) fluorophore. Anal Bioanal Chem 405, 2065–2075, 10.1007/s00216-012-6623-1 (2013). 23254457 10.1007/s00216-012-6623-1 PMC3566262

[b4] Bunzli J. C. G. & Eliseeva S. V. Intriguing aspects of lanthanide luminescence. Chem Sci 4, 1939–1949, 10.1039/C3sc22126a (2013).

[b5] Shinoda S. Dynamic cyclen-metal complexes for molecular sensing and chirality signaling. Chemical Society reviews 42, 1825–1835, 10.1039/C2cs35295h (2013). 23034678 10.1039/c2cs35295h

[b6] Cheng L., Wang C. & Liu Z. Upconversion nanoparticles and their composite nanostructures for biomedical imaging and cancer therapy. Nanoscale 5, 23–37, 10.1039/c2nr32311g (2013). 23135546 10.1039/c2nr32311g

[b7] Lichtman J. W. & Conchello J. A. Fluorescence microscopy. Nature methods 2, 910–919, 10.1038/nmeth817 (2005). 16299476 10.1038/nmeth817

[b8] Sagolla K., Lohmannsroben H. G. & Hille C. Time-resolved fluorescence microscopy for quantitative Ca2+ imaging in living cells. Anal Bioanal Chem 405, 8525–8537, 10.1007/s00216-013-7290-6 (2013). 23975087 10.1007/s00216-013-7290-6

[b9] Vaasa A. *et al.* Time-gated luminescence microscopy with responsive nonmetal probes for mapping activity of protein kinases in living cells. Chemical communications 48, 8595–8597, 10.1039/c2cc33565d (2012). 22822483 10.1039/c2cc33565d PMC3415249

[b10] Moore J. D., Lord R. L., Cisneros G. A. & Allen M. J. Concentration-independent pH detection with a luminescent dimetallic Eu(III)-based probe. Journal of the American Chemical Society 134, 17372–17375, 10.1021/Ja307098z (2012). 23067148 10.1021/ja307098z PMC3492840

[b11] Lechevallier S. *et al.* Luminescence properties of mesoporous silica nanoparticles encapsulating different europium complexes: application for biolabelling. J Nanomater, 10.1155/2013/918369 (2013).

[b12] Rich R. M. *et al.* Multiple-pulse pumping for enhanced fluorescence detection and molecular imaging in tissue. Methods, 10.1016/j.ymeth.2013.08.026 (2013). 10.1016/j.ymeth.2013.08.026 PMC3938978 23994243

[b13] Jin D. & Piper J. A. Time-gated luminescence microscopy allowing direct visual inspection of lanthanide-stained microorganisms in background-free condition. Analytical chemistry 83, 2294–2300, 10.1021/ac103207r (2011). 21344865 10.1021/ac103207r

[b14] Deng W. *et al.* Ultrabright Eu-doped plasmonic Ag@SiO2 nanostructures: time-gated bioprobes with single particle sensitivity and negligible background. Advanced materials 23, 4649–4654, 10.1002/adma.201102027 (2011). 21913234 10.1002/adma.201102027

[b15] Wu J. *et al.* Visible-light-sensitized highly luminescent europium nanoparticles: preparation and application for time-gated luminescence bioimaging. J Mater Chem 19, 1258–1264, 10.1039/B815999h (2009).

[b16] Duncan M. D., Mahon R., Tankersley L. L. & Reintjes J. Time-gated imaging through scattering media using stimulated raman amplification. Opt Lett 16, 1868–1870, 10.1364/Ol.16.001868 (1991). 19784165 10.1364/ol.16.001868

[b17] Requejo-Isidro J. *et al.* High-speed wide-field time-gated endoscopic fluorescence-lifetime imaging. Opt Lett 29, 2249–2251, 10.1364/Ol.29.002249 (2004). 15524370 10.1364/ol.29.002249

[b18] Soini A. E., Kuusisto A., Meltola N. J., Soini E. & Seveus L. A new technique for multiparameter imaging microscopy: use of long decay time photoluminescent labels enables multiple color immunocytochemistry with low channel-to-channel crosstalk. Microscopy research and technique 62, 396–407, 10.1002/jemt.10389 (2003). 14601145 10.1002/jemt.10389

[b19] Hanaoka K., Kikuchi K., Kobayashi S. & Nagano T. Time-resolved long-lived luminescence imaging method employing luminescent lanthanide probes with a new microscopy system. Journal of the American Chemical Society 129, 13502–13509, 10.1021/ja073392j (2007). 17927176 10.1021/ja073392j

[b20] Connally R., Jin D. & Piper J. High intensity solid-state UV source for time-gated luminescence microscopy. Cytometry. Part A: the journal of the International Society for Analytical Cytology 69, 1020–1027, 10.1002/cyto.a.20326 (2006). 16888769 10.1002/cyto.a.20326

[b21] Connally R. E. & Piper J. A. Time-gated luminescence microscopy. Annals of the New York Academy of Sciences 1130, 106–116, 10.1196/annals.1430.032 (2008). 18596339 10.1196/annals.1430.032

[b22] Hu B. L., He Y. H. & Liu Z. Y. NIR area array CCD-based singlet oxygen luminescence imaging for photodynamic therapy. J Phys Conf Ser 277, 10.1088/1742-6596/277/1/012011 (2011).

[b23] Ibanez G., McBean J. L., Astudillo Y. M. & Luo M. K. An enzyme-coupled ultrasensitive luminescence assay for protein methyltransferases. Analytical biochemistry 401, 203–210, 10.1016/J.Ab.2010.03.010 (2010). 20227379 10.1016/j.ab.2010.03.010

[b24] Lu Y. *et al.* Automated detection of rare-event pathogens through time-gated luminescence scanning microscopy. in Cytometry A Vol. 79, 349–355, 10.1002/Cyto.A.21045 (2011). 21462305 10.1002/cyto.a.21045

[b25] Jin D. Y. Demonstration of true-color high-contrast microorganism imaging for terbium bioprobes. Cytom Part A 79A, 392–397, 10.1002/Cyto.A.21052 (2011). 10.1002/cyto.a.21052 21448978

[b26] Jin D. Y., L Y. Q., Leif R. C., Yang S., Rajendran M. & Miller L. W. in Current Protocols in Cytometry How to build a time-gated luminescence microscope 67: 62.22:62.22.61–62.22.36. (2014). 10.1002/0471142956.cy0222s67 PMC13628505 24510771

[b27] Beverloo H. B., Vanschadewijk A., Vangelderenboele S. & Tanke H. J. Inorganic phosphors as new luminescent labels for immunocytochemistry and time-resolved microscopy. Cytometry 11, 784–792, 10.1002/cyto.990110704 (1990). 2272243 10.1002/cyto.990110704

[b28] Vereb G., Jares-Erijman E., Selvin P. R. & Jovin T. M. Temporally and spectrally resolved imaging microscopy of lanthanide chelates. Biophysical journal 74, 2210–2222, 10.1016/S0006-3495(98)77930-5 (1998). 9591648 10.1016/S0006-3495(98)77930-5 PMC1299564

[b29] Wu S. *et al.* Non-blinking and photostable upconverted luminescence from single lanthanide-doped nanocrystals. Proceedings of the National Academy of Sciences of the United States of America 106, 10917–10921, 10.1073/pnas.0904792106 (2009). 19541601 10.1073/pnas.0904792106 PMC2698891

[b30] Lu Y., Xi P., Piper J. A., Huo Y. & Jin D. Time-gated orthogonal scanning automated microscopy (OSAM) for high-speed cell detection and analysis. Sci. Rep. 2, 837, 10.1038/srep00837 (2012). 23150787 10.1038/srep00837 PMC3495287

[b31] Lu Y. *et al.* On-the-fly decoding luminescence lifetimes in the microsecond region for lanthanide-encoded suspension arrays. Nature Communications, 10.1038/ncomms4741 (2014). 10.1038/ncomms4741 PMC4024748 24796249

[b32] Lu Y. *et al.* Tunable lifetime multiplexing using luminescent nanocrystals. Nature Photonics 8, 33–37, 10.1038/nphoton.2013.322 (2014).

[b33] Yuan J. L., Wang G. L., Kimura H. & Matsumoto K. Highly sensitive time-resolved fluoroimmunoassay of human immunoglobulin E by using a new europium fluorescent chelate as a label. Analytical biochemistry 254, 283–287, 10.1006/abio.1997.2444 (1997). 9417790 10.1006/abio.1997.2444

[b34] Osseni S. A. *et al.* Gadolinium oxysulfide nanoparticles as multimodal imaging agents for T2-weighted MR, X-ray tomography and photoluminescence. Nanoscale 6, 555–564, 10.1039/c3nr03982j (2013). 24241248 10.1039/c3nr03982j

