# Supplementary material for: Practical Implementation, Characterization and Applications of a Multi-Colour Time-Gated Luminescence Microscope
Source: Sci Rep. 2014 Oct 13;4:6597. doi: 10.1038/srep06597 (PMC4194433; doi:10.1038/srep06597)
Supplement: Supplementary Information — Practical Implementation, Characterization and Applications of a Multi-Colour Time-Gated Luminescence Microscope [file srep06597-s1.doc]

**Practical Implementation, Characterization and Applications of a Multi-Colour Time-Gated Luminescence Microscope**

Lixin Zhang^1^, Xianlin Zheng^1^, Wei Deng^1^, Yiqing Lu^1*^, Severine Lechevallier^2^, Zhiqiang Ye^3^, Ewa Goldys^1^, Judith M. Dawes^1^, James A Piper^1^, Jingli Yuan^3^, Marc Verelst^2^, Dayong Jin^1*^

*^1^Advanced Cytometry Labs, ARC Centre of Excellence for Nanoscale BioPhotonics (CNBP), Macquarie University, Sydney, NSW 2109, Australia*

*^2^Centre d'Élaboration de Matériaux et d'Etudes Structurales (CERMES - CNRS), Paul Sabatier University, France*

*^3^State Key Laboratory of Fine Chemicals, School of Chemistry, D alian University of Technology, Dalian 116024, China*

^*^Email: [yiqing.lu@mq.edu.au](mailto:yiqing.lu@mq.edu.au); or: [dayong.jin@mq.edu.au](mailto:dayong.jin@mq.edu.au)

**S1. Xenon lamp excitation spectrum**

**Figure S1**. The excitation light from the xenon flash lamp has a spectral region from 320 nm to 400 nm, after passing through a UV band-pass filter (U-360, Edmund)

**S2. Modification of chopper blades**


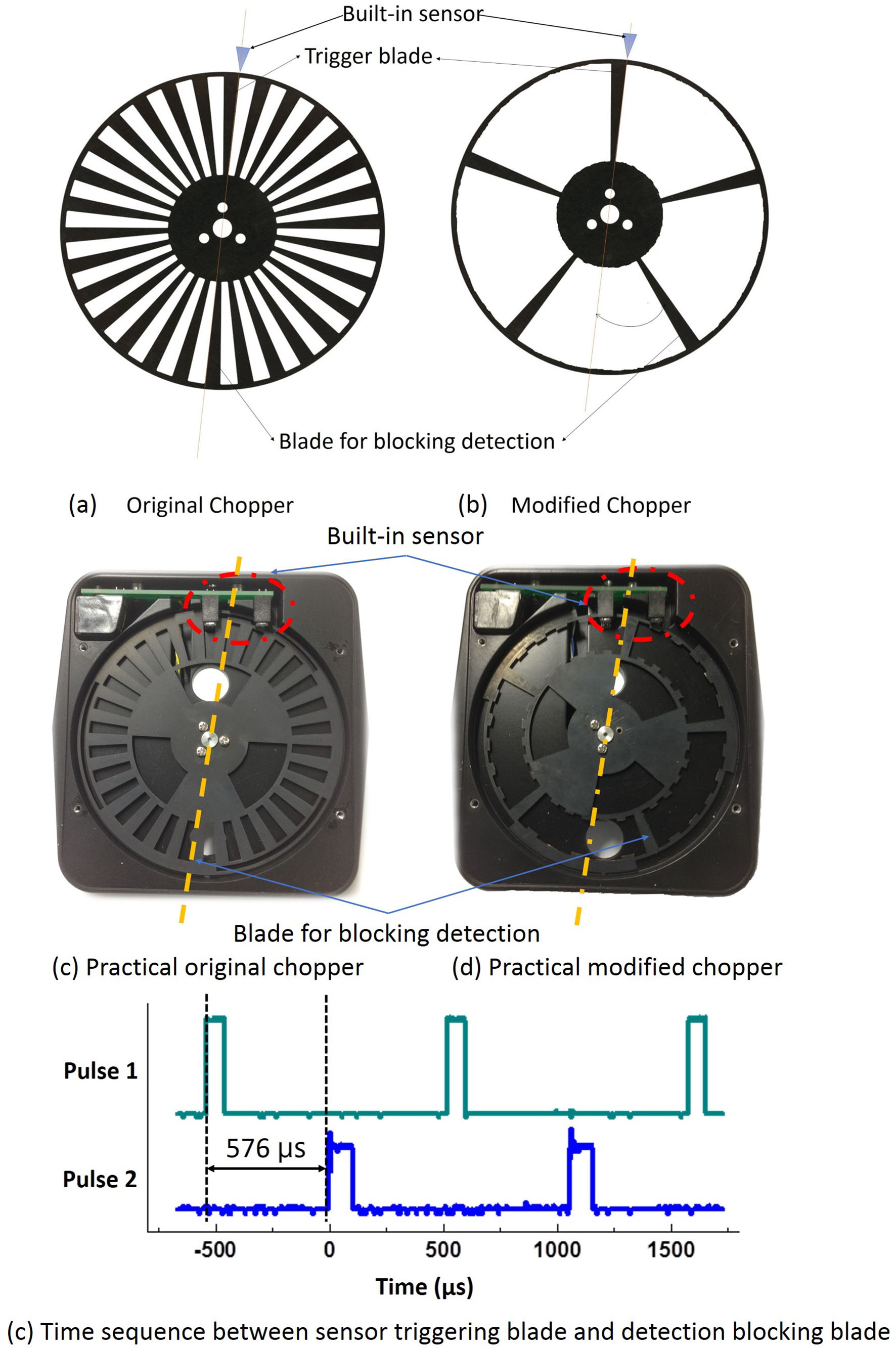


**Figure S2.** The induced time delay between the sensor trigger blade and the detection blocking blade in the modified chopper

**S3. Correction of optical distortion**


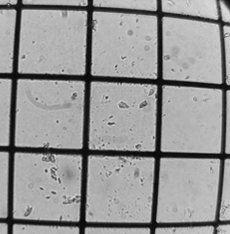

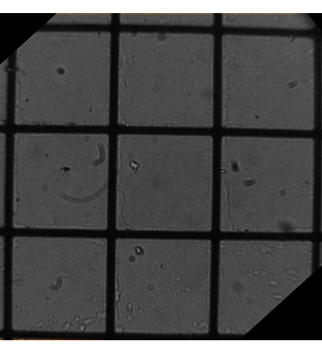


**Figure S3.** A wide-field image with barrel distortion (left), and its improved image without barrel distortion (right), after all optical components are aligned to be exactly coaxial. Each grid represents 50×50 µm^2^.

**S4. Image analysis to determine the signal-to-background ratio**

The average signal and background levels for the red and the green channels of the images captured under the non-time-gated mode and the time-gated mode were analysed based on the procedures illustrated below, with the two images in Figure S4 as the example. Matlab was used in this study, but other image processing software, such as ImageJ, can also be used.


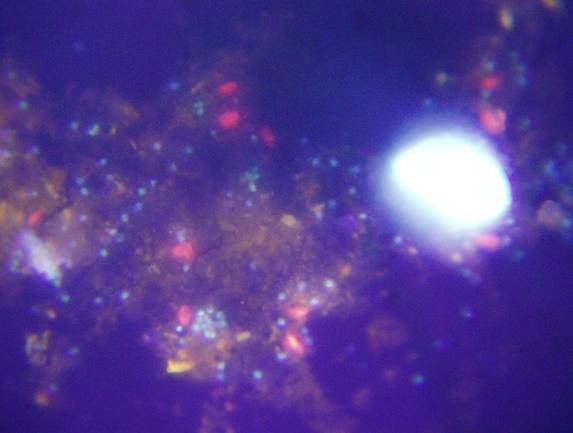

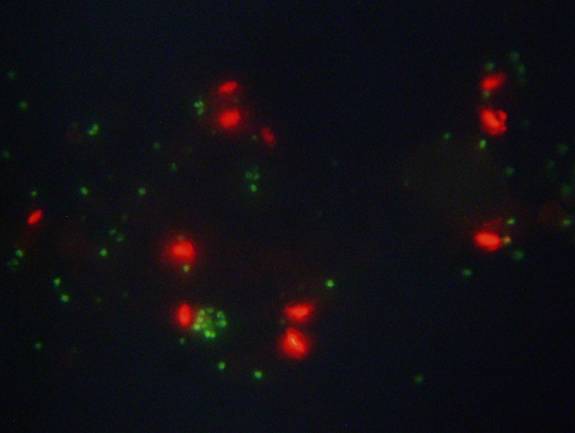


**Figure S4.** The non-time-gated (left) and time-gated (right) dual-colour images to be referred to in the image analysis demonstration.

**S4.1 Analysing signal levels in time-gated images**

The time-gated colour image was split into the red, green and blue channels. Figure S4.1-1 shows the monocolour images of the red and green channels, while the blue channel was not considered in the following analysis.


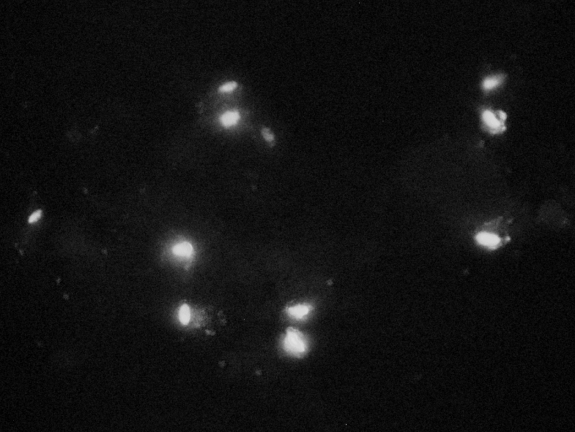

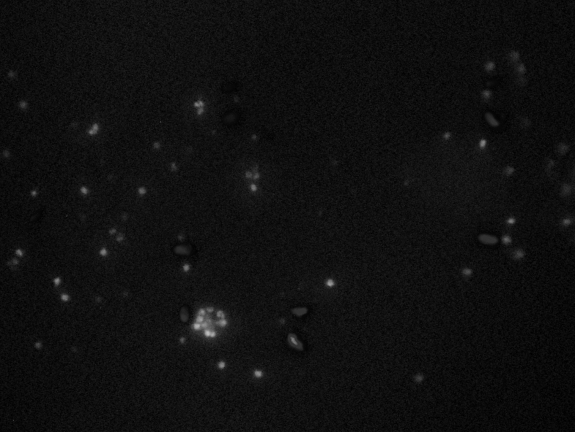


**Figure S4.1-1** The red (left) and green (right) channels of the time-gated image.

Image masks with thresholds equal to 0.3 times the maximum intensities in respective channels were applied on these monocolour images to effectively select target cells, as shown in Figure S4.1-2.


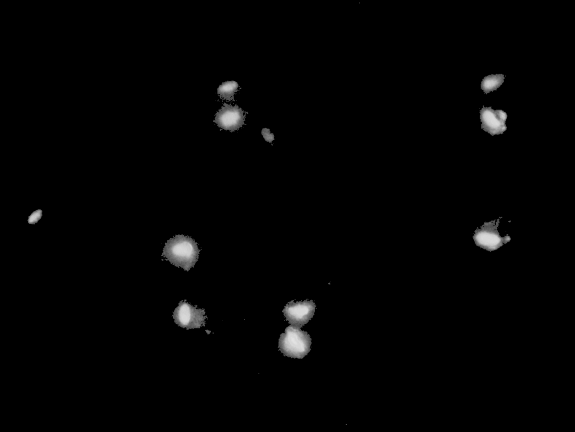

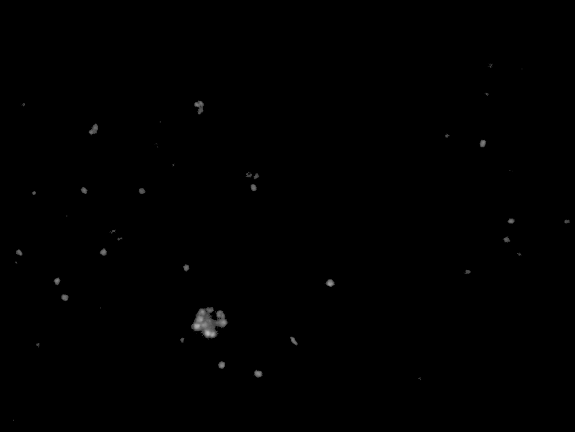


**Figure S4.1-2** The time-gated monocolour images with masks to select target cells, for the red channel (left) and the green channel (right).

The maximum, minimum and average signal intensities were obtained for the masked areas.

**S4.2 analysing background levels in time-gated images**

The above masks were reversed and then applied to the images in Figure S4.1-1 again, to select the non-target areas, as shown in Figure S4.2, before the maximum, minimum and average background intensities were calculated.


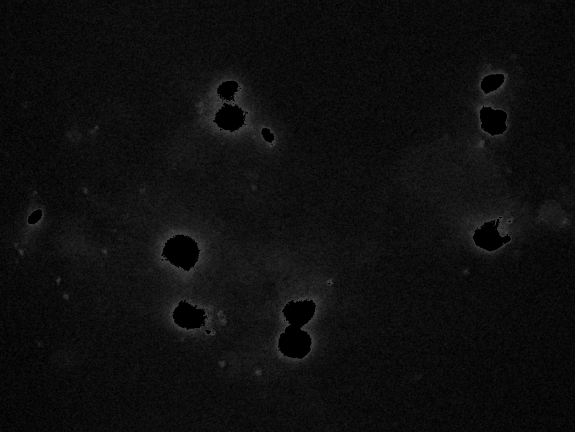

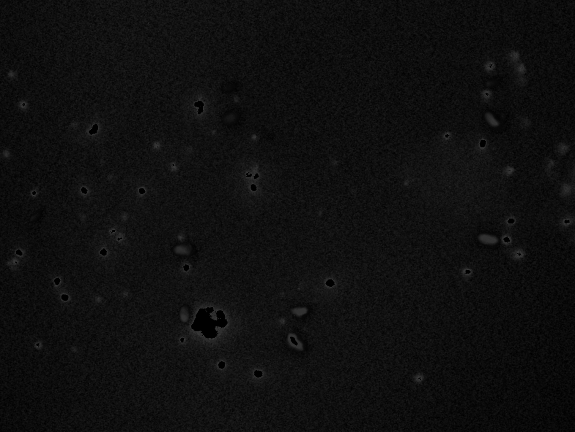


**Figure S4.2** The time-gated monocolour images with background areas masked for the red channel (left) and the green channel (right).

**S4.3 Analysing signal levels in non-time-gated images**

The non-time-gated colour image was split into the red, green and blue channels, as shown in Figure S4.3-1 (again, the blue channel was ignored).


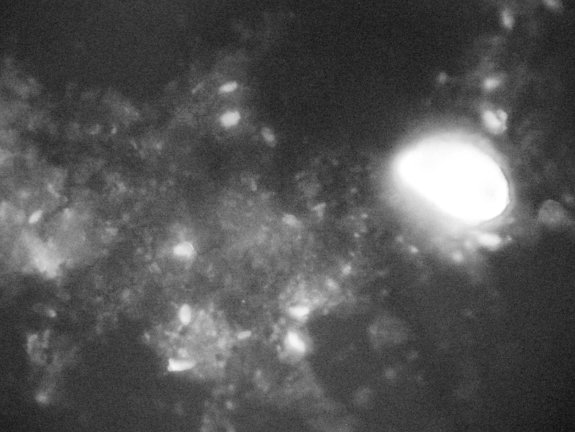

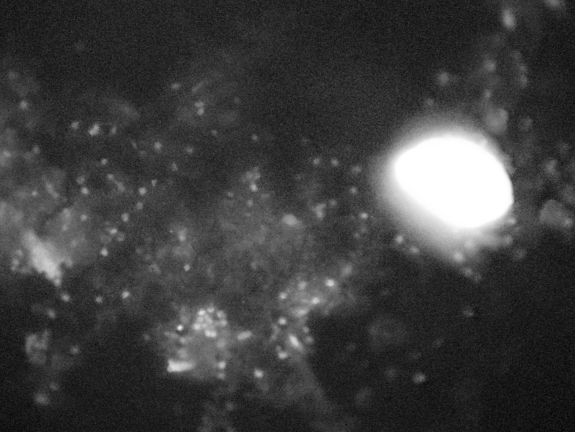


**Figure S4.3-1** The red (left) and green (right) channels of the non-time-gated image.

The same masks used in S4.1 were applied to select the areas that contained target cells, and the maximum, minimum and average signal intensities were calculated.


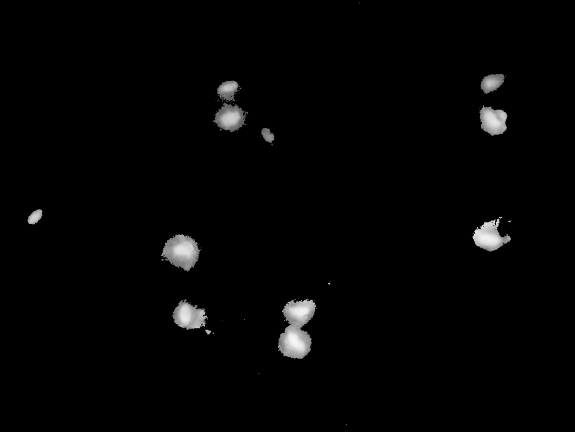

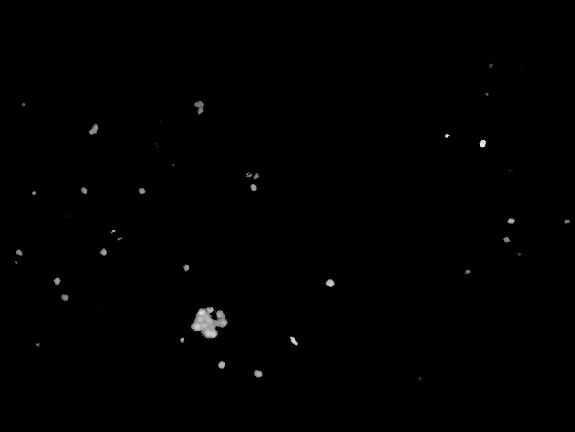


**Figure S4.3-2.** The non-time-gated monocolour images with masks selecting target cells, for the red channel (left) and the green channel (right).

**S4.4 Analysing background levels in non-time-gated images**

The same masks used in S4.2 were applied to the images in Figure S4.3-1, to select the non-target areas, as shown in Figure S4.4, before the maximum, minimum and average background intensities were calculated.


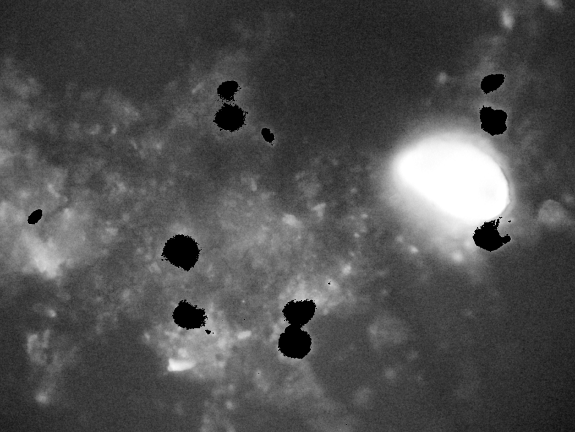

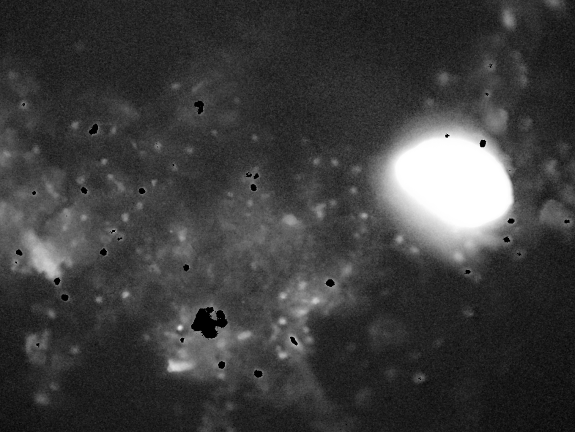


**Figure S4.4.** The non-time-gated monocolour images with background areas masked for the red channel (left) and the green channel (right).

**Table S1. Signal and background levels of the red channel for ten pairs of images under non-time-gated and time-gated modes.**

| Luminescence from Eu complexes measured in the red channel | | | | | | | |
| --- | --- | --- | --- | --- | --- | --- | --- |
|  | Image No. | S_max | S_min | S_avg | B_max | B_min | B_avg |
| Non-time-gated mode | 1 | 252 | 75 | 167 | 255 | 11 | 82 |
|  | 2 | 221 | 74 | 147 | 183 | 14 | 51 |
|  | 3 | 168 | 42 | 105 | 126 | 0 | 29 |
|  | 4 | 239 | 63 | 150 | 254 | 20 | 81 |
|  | 5 | 219 | 37 | 112 | 107 | 0 | 26 |
|  | 6 | 217 | 31 | 112 | 182 | 0 | 42 |
|  | 7 | 219 | 34 | 122 | 109 | 0 | 23 |
|  | 8 | 219 | 38 | 114 | 118 | 0 | 28 |
|  | 9 | 226 | 34 | 129 | 173 | 3 | 42 |
|  | 10 | 208 | 35 | 105 | 128 | 2 | 33 |
| Time-gated mode | 1 | 228 | 69 | 133 | 68 | 0 | 15 |
|  | 2 | 220 | 67 | 130 | 66 | 0 | 10 |
|  | 3 | 205 | 62 | 133 | 61 | 0 | 8 |
|  | 4 | 222 | 67 | 144 | 66 | 0 | 12 |
|  | 5 | 220 | 67 | 137 | 66 | 0 | 11 |
|  | 6 | 221 | 67 | 141 | 66 | 0 | 9 |
|  | 7 | 220 | 67 | 147 | 66 | 0 | 8 |
|  | 8 | 222 | 67 | 142 | 66 | 0 | 12 |
|  | 9 | 222 | 67 | 132 | 66 | 0 | 14 |
|  | 10 | 222 | 67 | 127 | 66 | 0 | 11 |

**Table S2.** Signal and background levels of the green channel for ten pairs of images under non-time-gated and time-gated modes.

| Luminescence from Tb complexes measured in the green channel | | | | | | | |
| --- | --- | --- | --- | --- | --- | --- | --- |
|  | Image No. | S_max | S_min | S_avg | B_max | B_min | B_avg |
| Non-time-gated mode | 1 | 252 | 46 | 144 | 255 | 11 | 84 |
|  | 2 | 221 | 38 | 101 | 213 | 14 | 52 |
|  | 3 | 108 | 23 | 71 | 168 | 0 | 29 |
|  | 4 | 241 | 57 | 190 | 254 | 20 | 82 |
|  | 5 | 102 | 23 | 58 | 219 | 0 | 27 |
|  | 6 | 217 | 18 | 79 | 217 | 0 | 43 |
|  | 7 | 131 | 17 | 41 | 219 | 0 | 24 |
|  | 8 | 219 | 19 | 64 | 219 | 0 | 30 |
|  | 9 | 226 | 16 | 107 | 226 | 3 | 44 |
|  | 10 | 103 | 18 | 58 | 208 | 2 | 34 |
| Time-gated mode | 1 | 194 | 59 | 91 | 58 | 0 | 15 |
|  | 2 | 111 | 34 | 45 | 33 | 0 | 11 |
|  | 3 | 178 | 54 | 80 | 53 | 0 | 9 |
|  | 4 | 114 | 35 | 47 | 34 | 0 | 11 |
|  | 5 | 206 | 62 | 91 | 61 | 0 | 10 |
|  | 6 | 184 | 56 | 74 | 55 | 0 | 9 |
|  | 7 | 181 | 55 | 66 | 54 | 0 | 11 |
|  | 8 | 188 | 57 | 78 | 56 | 0 | 11 |
|  | 9 | 179 | 54 | 73 | 53 | 0 | 11 |
|  | 10 | 189 | 57 | 89 | 56 | 0 | 10 |

**S5. Imaging results under UV LED excitation**

**
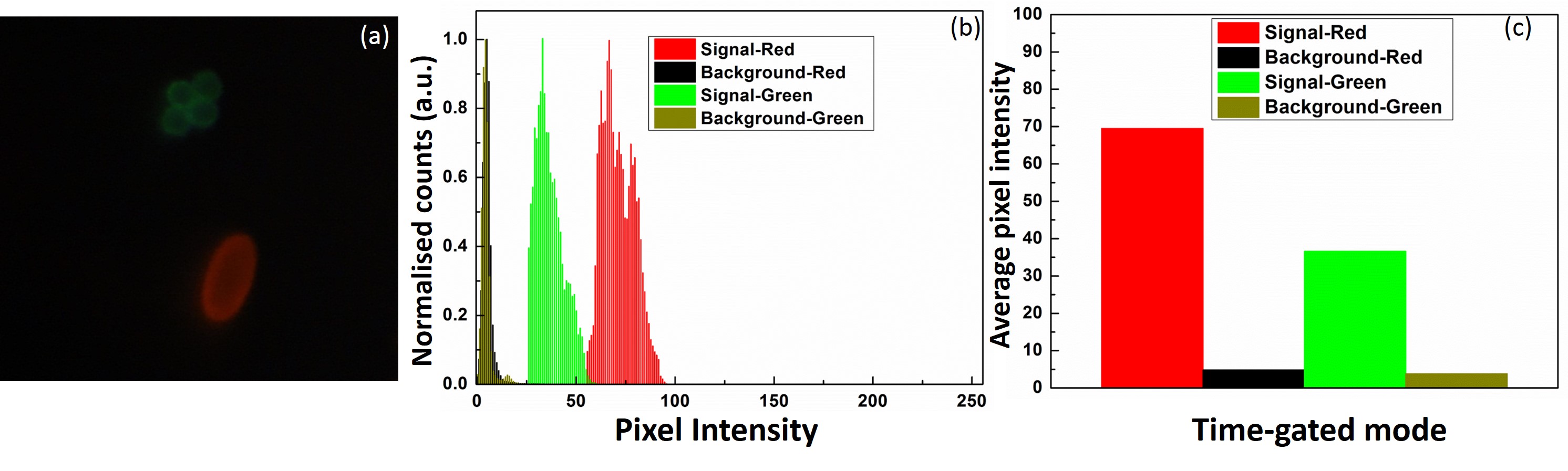
**

**Figure S5.** Imaging results under UV LED excitation. (a) Dual-colour time-gated image of Eu-labelled *Giardia* and Tb-labelled *Cryptosporidium*, with an exposure time of 15 seconds. (b) Pixel intensity histograms of the signal and background levels in the red and green channels. (c) Bar chart showing the average signal and background levels. The signal-to-background ratios are 70:5.6 for the red channel and 37:4.2 for the green channel.

**S6. Calculation of the crosstalk**

In addition to time gating, accurate quantification of the luminescence intensities for the two pathogens requires calibration of the crosstalk between the red and green channels, which is caused by the satellite emission peaks of the Eu and Tb complexes.

The relative responsivity curves for the red, green and blue channels of the Olympus DP71 camera can be found in its user manual available online, as shown in Figure S6.1. The curves were digitized using the GetData Graph Digitizer software.


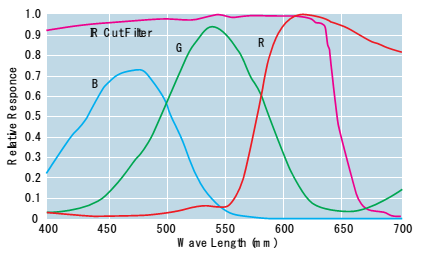


**Figure S6.1** Relative responsivity curves for the Olympus DP71 camera.

**Figure S6.2** Emission spectra of the Eu and Tb complexes.

The emission spectra of the Eu and Tb complexes, as shown in Figure S11, were multiplied by the three responsivity curves, respectively, followed by normalisation, to calculate their contribution proportions to the three colour channels. The results are given in Table S3.

**Table S3. The contributions of the Eu and Tb emission to the colour channels of the camera.**

|  | Red Channel | Green Channel | Blue Channel |
| --- | --- | --- | --- |
| Eu emission | 78.6% | 13.6% | 7.8% |
| Tb emission | 19.2% | 59.9% | 20.9% |

**S7. Co-localization analysis of the nanoparticle images**

The time-gated luminescence image of the Eu nanoparticles and its corresponding TEM image were compared using the colocalization function in the ImageJ software. The images were first resized to ensure they covered the identical area, before transformed into 8-bit grey-scale, as shown in Figure S7.


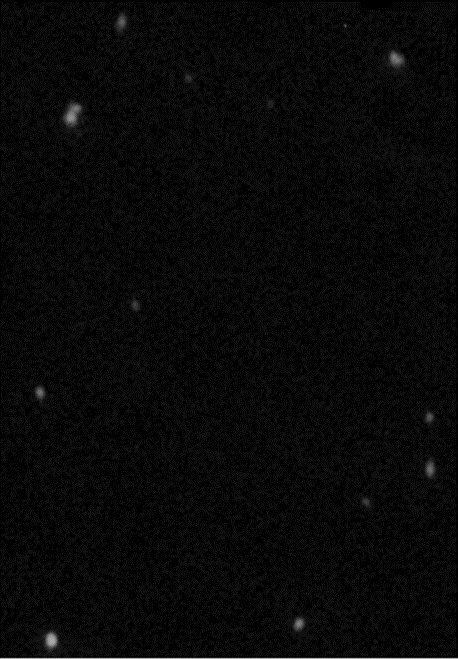

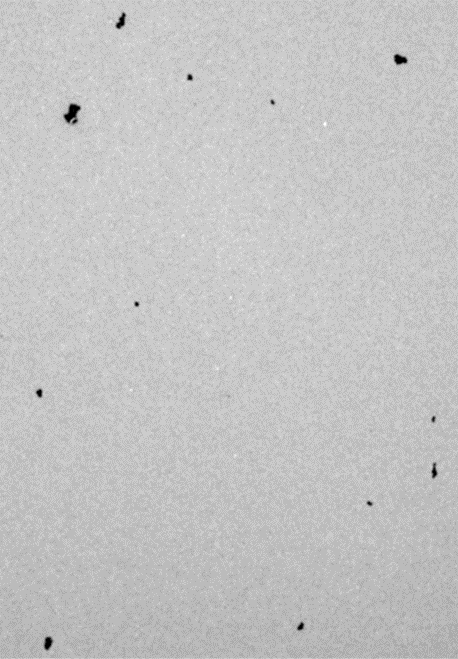


**Figure S7.** Grey-scale images of the original time-gated luminescence image (left) and the TEM image (right) of the Eu nanoparticles.

They were imported into the colocalization plugin to generate the co-localisation image, as shown in Figure 4d in the main text.
